# Health system governance to support integrated mental health care in South Africa: challenges and opportunities

**DOI:** 10.1186/s13033-015-0004-z

**Published:** 2015-03-11

**Authors:** Debra Leigh Marais, Inge Petersen

**Affiliations:** EMERALD project, School of Applied Human Sciences, University of KwaZulu-Natal, P/Bag X01, Scottsville, Pietermaritzburg, 3201 South Africa; EMERALD project, School of Applied Human Sciences, University of KwaZulu-Natal, Durban, South Africa

**Keywords:** Mental health system, Integrated care, Governance, Barriers & facilitative factors, South Africa

## Abstract

**Background:**

While South Africa has a new policy framework supporting the integration of mental health care into primary health care, this is not sufficient to ensure transformation of the health care system towards integrated primary mental health care. Health systems strengthening is needed, incorporating, *inter alia*, capacity building and resource inputs, as well as good governance for ensuring that the relevant policy imperatives are implemented.

**Objectives:**

To identify systemic factors within institutional and policy contexts that are likely to facilitate or impede the implementation of integrated mental health care in South Africa.

**Methods:**

Semi-structured qualitative interviews were conducted with 17 key stakeholders in the Department of Health and Department of Social Development at national level, at provincial level in the North West Province, and at district level in the Dr Kenneth Kaunda district. Participants were purposively identified based on their positions and job responsibilities. Interview questions were guided by a hybrid of Siddiqi et al.’s governance framework principles and Mikkelsen-Lopez et al.’s health system governance approach. Data were analysed using framework analysis in *NVivo*.

**Results:**

Facilitative factors included the recent mental health care policy framework and national action plan that embraces integrated care using a task sharing model and provides policy imperatives for the establishment of district mental health teams to facilitate the development and implementation of district mental health care plans; the roll out of the integrated chronic disease service delivery platform that can be leveraged to increase access and resources as well as decrease stigma; and the presence of NGOs that can assist with service delivery. Challenges included the low prioritisation and stigmatisation of mental illness; weak managerial and planning capacity to develop and implement mental health care plans at provincial and district level; poor pre-service training of generalists in mental health care; weak orientation to integrated care; high staff turnover; weak intersectoral coordination; infrastructural constraints; and no dedicated mental health budget.

**Conclusion:**

This study identifies strategies to support and improve integrated mental health care in primary health care services.

## Background

Lifestyle changes and better control of infectious diseases have resulted in an epidemiological transition from communicable to noncommunicable diseases (NCDs). By 2030, NCDs, including neuropsychiatric disorders, will constitute seven of the top ten causes of disease burden globally, with depression predicted to be the leading cause of disease burden [1]. Coupled with the transition of communicable diseases such as HIV/AIDS to chronic conditions, there is a need for health care systems to increasingly provide chronic care. Chronic diseases, including mental disorders, frequently co-exist with one another [2-4] and the relationship between physical and mental disorders is bi-directional. Not only does mental illness affect the prognosis (course and outcome) of other chronic conditions in terms of help-seeking, diagnosis, quality of care provided, treatment, and adherence [4]; many health conditions increase the risk for mental disorder [2,4,5] resulting in greater burden on the health care system and poorer patient outcomes. Integrating mental health into chronic care services, particularly at the primary health care level, is likely to lead to improved medication adherence and lower healthcare costs in low- and middle-income countries (LAMICs) [6], with integrated collaborative care for clusters of coexisting illnesses, especially physical and mental disorders, increasingly shown to be more cost-effective than usual care in high income countries [7]. There is growing evidence that integrating mental health into primary care is a viable way of treating common mental disorders, including depression and alcohol abuse [8-10]. However, while integration underpins the World Health Organization’s Mental Health Action Plan (2013–2020) [11], actions to integrate mental health is slow. Mental health is still a low priority in many LAMICs [12], with over two thirds of those affected by mental illness worldwide not in receipt of the care they need [13].

Like many other countries, South Africa is currently experiencing a rising burden of chronic conditions. The country suffers from a quadruple burden of disease: HIV/AIDS contributes the largest burden, followed by NCDs, other communicable and maternal/perinatal and nutritional conditions, and injuries [14]. The health system's response to the NCD burden has taken a back seat [14], partly due to the demands placed on the system by the overwhelming HIV/AIDS pandemic [15]. Competing with multiple pressing health concerns, resource allocation for mental illness in South Africa follows the global trend of being insufficient, inequitably distributed and inefficiently utilised in relation to need [16-19]. Nonetheless, compared to many other African countries, South Africa appears to be relatively well resourced in terms of mental health facilities, human resources, and provision of psychotropic medications [17]. It also appears to have made progress towards decentralised care, particularly with respect to secondary care, with designated hospitals providing a 72-hour emergency management and observation service. However, integration into the primary health care system still remains a challenge [18] and three out of four people with a common mental disorder are not in receipt of any care [20].

South Africa’s progressive Mental Health Care Act was adopted in 2002. The Mental Health Care Act was seen as an important step towards addressing mental health as a public health issue [21], as well as advancing the human rights of those requiring mental health care, including the right to access to care [22]. However, it has not been without its challenges, particularly with respect to implementation and enforcement in an already over-burdened health system [16,21]. In 2013, South Africa adopted a new Mental Health Policy Framework (MHPF) and Strategic Plan 2013–2020 [23] aligned to the WHO Mental Health Action plan that embraces task sharing and the integration of mental health into primary health care services. The MHPF is widely regarded as South Africa's first official mental health policy and is an important tool for the implementation of the Mental Health Care Act of 2002 [17,24]. It integrates scientific evidence and best practice with an emphasis on human rights and vulnerable populations [22]. The Strategic Plan 2013–2020 outlines eight key objectives: i) district based mental health services and integration of mental health into primary health care; ii) institutional capacity building for mental health care; iii) surveillance, research and innovation to strengthen the quality of services; iv) strengthening infrastructure and capacity of facilities to provide mental health care; v) improving mental health technology, equipment and medicines; vi) deepening inter-sectoral collaboration; vii) capacitating human resources for mental health; viii) advocacy, mental health promotion and prevention of mental illness.

In response to the growing burden of chronic conditions, the South African National Department of Health has also introduced a multi-disease integrated chronic disease management model at the primary health care facility, community and population levels [25]. This model uses a health system building blocks approach involving: 1) health service re-organisation at facility level, 2) clinical management support at facility level, 3) assisted self-management support at community level, and 4) strengthening of support systems and structures within the health system [25]. This integrated chronic disease system has the potential to provide an enabling platform for the integration of mental health into primary health care. Its stepped care approach accounts for the fact that, while primary care for mental health is a critical component of general primary health care, it is not sufficient to address the full spectrum of mental health needs of the population [10]. However, optimal functioning of these inter-related elements is dependent on overarching strong stewardship and ownership at all levels of the health system.

Governance inputs and processes underscore health system performance, from providing strategic direction through policy frameworks to managing policy implementation through system design. Some argue that, despite its growing visibility in global health debates, the concept of governance – and how it differs from management – is still poorly understood [26]. Perhaps as a result of this, there are few frameworks or approaches to systematically assess governance in health systems [27]. Health governance concerns “the actions and means adopted by a society to organize itself in the promotion and protection of the health of its population” [28]. It thus goes beyond the formal mechanisms of government to include the “totality of ways in which a society organises and collectively manages its affairs” [26]. For this paper, we have used the WHO [29] definition of governance as “ensuring strategic policy frameworks exist and are combined with effective oversight, coalition-building, the provision of appropriate regulations and incentives, attention to system- design, and accountability” (p. vi). Management is also part of governance in so far as it is concerned with implementing policies and decisions [27].

### Health system governance framework

Recognising that governance is the least well understood aspect of the health system, Siddiqi and colleagues [30] developed a framework for the assessment of health system governance at national and sub-national levels. The framework permits “diagnoses of the ills” in health system governance at the policy and operational levels, and points to interventions for its improvement [30]. Drawing on a number of existing good governance and stewardship principles, the framework identifies ten principles for assessing governance and defines domains against which these principles can be measured across different levels of the system. These principles are: rule of law; strategic vision; participation & consensus orientation; transparency; responsiveness; equity; effectiveness & efficiency; accountability; intelligence & information; and ethics. Definitions for these principles, and their associated domains, are provided in Table 1.

**Table 1:** **Siddiqi et al.’s** [30] **governance framework principles**

**Principle**	**Domains**
**Strategic vision**	
Leaders have a broad and long-term perspective on health and human development, along with a sense of strategic directions for such development. There is also an understanding of the historical, cultural and social complexities in which that perspective is grounded	Long-term vision; comprehensive development strategy including health
**Participation & consensus orientation**	
All men and women should have a voice in decision-making for health, either directly or through legitimate intermediate institutions that represent their interests. Such broad participation is built on freedom of association and speech, as well as capacities to participate constructively. Good governance of the health system mediates differing interests to reach a broad consensus on what is in the best interests of the group and, where possible, on health policies and procedures	Participation in decision-making process; stakeholder identification and voice
**Rule of law**	
Legal frameworks pertaining to health should be fair and enforced impartially, particularly the laws on human rights related to health	Legislative process; interpretation of legislation to regulation and policy; enforcement of laws and regulations
**Transparency**	
Transparency is built on the free flow of information for all health matters. Processes, institutions and information should be directly accessible to those concerned with them, and enough information is provided to understand and monitor health matters	Transparency in decision-making; transparency in allocation of resources
**Responsiveness**	
Institutions and processes should try to serve all stakeholders to ensure that the policies and programs are responsive to the health and non-health needs of its users	Response to population health needs; response to regional local health needs
**Equity**	
All men and women should have opportunities to improve or maintain their health and well-being	Equity in access to care; fair financing of health care; disparities in health
**Effectiveness & efficiency**	
Processes and institutions should produce results that meet population needs and influence health outcomes while making the best use of resources	Quality of human resources; communication processes;
capacity for implementation	
**Accountability**	
Decision-makers in government, the private sector and civil society organizations involved in health are accountable to the public, as well as to institutional stakeholders. This accountability differs depending on the organization and whether the decision is internal or external to an organization	Accountability: internal; accountability: external
**Intelligence & information**	
Intelligence and information are essential for a good understanding of health system, without which it is not possible to provide evidence for informed decisions that influences the behaviour of different interest groups that support, or at least do not conflict with, the strategic vision for health	Information: generation, collection, analysis, dissemination
**Ethics**	
The commonly accepted principles of health care ethics include respect for autonomy, nonmaleficence, beneficence and justice. Health care ethics, which includes ethics in health research, is important to safeguard the interest and the rights of the patients	Principles of bioethics; health care and research ethics

(Adapted from Siddiqi et al. [30])

**Figure 1:**
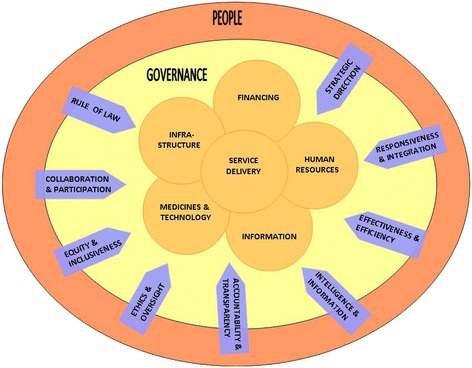
**A systemic approach to health system governance.**

While the goal of addressing governance challenges is to improve health system performance, Mikkelsen-Lopez et al. [27] recognise that improvements in governance may not necessarily result in improvements in overall health system performance due to various non-governance factors. It is therefore important to recognise the inter-relationships between governance and health system processes. Identifying and addressing barriers to health system performance is likely to improve governance, and vice versa. In Figure 1, we combine Siddiqi et al.’s [30] governance principles, adapted for the South African context, with Mikkelsen-Lopez et al.’s [27] health system governance approach to demonstrate the interplay between health system performance and governance of the system. While governance has been identified as one element of the health system [29], it can also be viewed as overarching, with governance inputs required for all levels of the system. This is consistent with Siddiqi et al.’s [30] assessment of governance across all levels of the health system.

South Africa’s new policy framework supporting the integration of mental health care into primary health care is not sufficient to ensure integrated primary mental health care as the service delivery platform into which mental health is being integrated needs to be functioning adequately as a first step. Assessing health system governance using the above approach allows for constraints on health system functioning to be identified and strategies developed to strengthen governance and functioning of the system as a whole. The aim of this study was thus to identify systemic factors within institutional and policy contexts that are likely to facilitate or impede the implementation of integrated mental health care in South Africa. This was done with the view to recommending strategies for strengthening the health system to support current legislature and policy imperatives for integrated care. This study forms part of the broader EMERALD programme (Emerging mental health systems in low- and middle-income countries) that is a research consortium of six low- and middle-income countries (Ethiopia, India, Nepal, Nigeria, South Africa, and Uganda) that aims to strengthen integrated mental health services in LAMICs through generating evidence of how best to strengthen health system performance to support integrated mental health care [31].

## Methods

### Design

A descriptive qualitative approach was adopted given the exploratory nature of the study. In particular, the study was guided by a framework analysis approach [32] given the descriptive nature of the study. Framework analysis is also used in policy-related research to make recommendations for systems and service improvements. The approach has been used for over 25 years and has recently become a popular method for primary qualitative health research, particularly in multi-disciplinary research teams [33]. Framework analysis is best applied to research that has specific questions, a limited time frame, a predetermined sample and a priori issues, where the primary task is to describe and interpret what is happening in a particular setting [34]. Because the framework analysis method is not aligned with a specific epistemological, philosophical or theoretical approach, it is a flexible tool that can be used in qualitative research that aims to generate themes [33].

### Recruitment and participants

Purposive sampling was used to recruit managers and policy makers at national, provincial and district levels on the basis that they could act as key informants regarding integration of mental health care into primary health care services. The target population was policy makers at the national level in the Department of Health, provincial (North West Province) coordinators and planners in primary health care and in mental health, and district-level (Dr Kenneth Kaunda district) managers of primary health care and mental health care services. The Dr Kenneth Kaunda district was chosen in the North West Province as it is the site of EMERALD activities and is a pilot site for the implementation of the integrated chronic disease management (ICDM) model.

Between January and April 2014, twenty four people were contacted to request their participation in this study. Seventeen of those 24 people were interviewed, yielding a response rate of 71%. Seventeen interviews were conducted with key informants at national (n = 4), provincial (n = 5) and district level (n = 8), within the government Department of Health (n = 14) and Department of Social Development (n = 2), as well as non-governmental organisations partnering with either of these two Departments (n = 1). The majority of participants were female (n = 12). Of note, the majority of the non-responders were at provincial level.

### Data collection

Data collection involved semi-structured qualitative interviews. The interview questions were guided by Siddiqi et al.’s governance framework [30], adapted for relevance to the current mental health context in South Africa as we move towards implementing the new mental health policy framework for integrated mental health care. In addition, the emphasis on health system functioning required by the implementation of this policy framework necessitated taking a systemic approach in interview questions, which was informed by Mikkelsen-Lopez et al.’s [27] health system governance approach. The interviews were audiotaped and transcribed verbatim.

### Data analysis

The interviews were analysed with a framework approach using the *NVivo* software programme. Framework analysis is a form of thematic analysis used to structure and summarise data in matrix form to identify commonalities and differences in the data to aid in the search for explanation and interpretation [33]. It is most suitable for analysis of interview data, where it is desirable to generate themes by making comparisons within and between cases [33]. It has also been adapted as a “best-fit” framework-based synthesis method where, as in the case of this study, a conceptual model suitable for the research question is used as the basis of the initial coding framework [35,36]. A combination of inductive and deductive coding was used in this study. An initial coding framework was developed, guided by a hybrid of Siddiqi et al.’s [30] governance framework principles and Mikkelsen-Lopez et al.’s [27] health system governance approach. The framework focused on barriers and facilitative factors to health system governance with respect to integrated mental health care. As analysis progressed, sub-themes emerging from the data were coded inductively, and added to the coding framework, while keeping the categories from the governance framework as parent themes.

### Ethics

Permission to conduct the interviews was obtained from the Department of Health at national and provincial levels. Ethical approval for the study was granted by the Biomedical Research Ethics Committee (BREC) at the University of KwaZulu-Natal (approval number BE407/13). Only participants who indicated that they understood the nature of the research and gave their voluntary informed consent were recruited into the study. The data collection methodologies are considered to represent minimal risk to the participants who agreed to participate in the studies. Structured, multi-level precautions were taken to safeguard the confidential nature of the information gathered, and to ensure the anonymity of the respondents, from data collection to data storage, analysis and publication. Research staff were trained to understand and implement ethical and research governance safeguards and protections. All participants were allocated a unique identifier code and the master identifier list kept in a password protected repository. Data and participant related files are subject to password-protected access.

## Results

### Rule of Law

This principle pertains to mental health legislation, including the enforcement of such legislation and the synergy of laws with mental health policies. While participants agreed that there were synergies between the Mental Health Care Act of 2002 and the new mental health policy framework of 2013 in terms of an emphasis on integration of mental health into primary health care, a key challenge was implementation at an operational level. Representatives from the Department of Social Development, in particular, felt that there was insufficient training on the Act, as well as a lack of clarity on responsibilities of the different sectors. Guidance on servicing people with intellectual disabilities also emerged as a pressing need: “There was no guidance and no document that would guide and actually talk to the responsibilities of Departments, on the provision of services to people with intellectual disabilities” (NR4). Suggested strategies for addressing these challenges included greater clarification in relation to roles and responsibilities with respect to the Act, particularly across sectors, as well as additional training on the Act to facilitate implementation and enforcement.

### Strategic Direction

Originally strategic vision, we amended this principle to strategic direction to encompass both the vision and the strategic policies and plans developed at national, provincial and district levels to integrate mental health into primary health care. In particular, we explored the development and implementation of policies and plans.

With regard to challenges, communication about the policy framework had purportedly not filtered down sufficiently to district level. One district level representative explained: “there wasn’t such in depth discussion on the document which I think is still lacking and we still have to do that so that everyone is on board” (DR1). There also seems to be poor coordination in terms of planning between national, provincial and district levels.

Insufficient capacity as a result of staff shortages and skills deficits to translate policies into plans also emerged as a key reason for the variation and poor quality of plans at provincial and district level. As suggested by a national representative,

“often these things that we do at the top, at national government, they’re always good on paper, and they sometimes arrive at the sites where they’re supposed to be implemented, and land up in cupboards. They gather dust…there’s a problem in resourcing this process” (NR2).

Lacking qualified managerial staff who could push implementation at ground level was a particular challenge in this respect. Problems with implementation were also attributed to inadequate resources and facilities at operational level.

Strategies to strengthen provincial and district implementation included support and constant communication from the national office, to ensure that the relevant managers understand the policy conceptually. Including strategic planners, who are responsible for the development of overall health plans, in developing mental health plans was seen as crucial. As noted by one participant,

“we are of the opinion that the strategic planners are better placed to make sure that mental health is included in their annual provincial plans and also, they develop the relevant indicators to make sure that the programme is monitored and evaluated” (NR3).

It was also suggested that greater clarity on the roles and responsibilities of different stakeholders across national, provincial and district levels with respect to implementation, would aid implementation, as would addressing resource and capacity disparities between provinces. Champions who can communicate well and “win people to their side” (NR2) were reportedly needed to advocate for mental health at provincial and district levels and to push policy implementation. In addition, building capacity in change management, particularly with facility managers, was identified as important to facilitate the implementation of collaborative chronic care at facility level. The use of district mental health teams in a public health role to coordinate the integration of mental health into district health plans was also endorsed, provided they are sufficiently capacitated.

### Responsiveness & Integration

In South Africa, this principle was amended to emphasise integration given the need for an enabling service delivery platform for integration. First, we explored responsiveness in terms of the public health priority of mental illness. Second, responsiveness to integration was explored at both facility and community levels. At facility level, this included consideration of barriers to integration, chronic care and rolling out Primary Care 101 (PC101), as well as the factors which facilitated these processes, such as the benefits of chronic care in terms of addressing comorbid conditions. Third, responsiveness to integration at community level was also explored.

#### Responsiveness to mental health as a public health priority

While one participant indicated that mental health was a priority at a political level and that there was political will and ministerial support at a national level as reflected in the new Mental Health Policy Framework and Strategic Plan, there was a general view that mental health was still not a priority in the face of many other health needs in South Africa, and was exacerbated by mental health being viewed separately from overall health:

“If you are HIV positive or you are AIDS sufferer, the mental health issue comes into play and it needs to be taken care of. If you suffer from diabetes or cancer, there are mental health issues. That’s how we need to understand it across the board, and my view is that we currently don’t understand it that way. Because we keep on saying it cuts across and I don’t think people understand when we say it cuts across” (PR4).

Some felt that other programmes – particularly communicable diseases like HIV and AIDS – have received all the attention at the expense of mental health as reflected in the following quotations from district and national representatives: “(Mental health) is still the stepchild of medicine to a great extent” (DR5).

“I don’t think it’s because they don’t think that mental health is important, but they do think that other things are more important… I also think that when people are dying in large numbers, health representatives feel that they need to respond to that, and who am I to say that they’re wrong? I think they’re probably right actually. So, you know, we face crises after crises” (NR1).

#### Responsiveness at a facility level

South Africa is piloting an integrated chronic disease management (ICDM) model in three districts, one being the Dr Kenneth Kaunda district in the North West Province. In this model, services for patients with chronic conditions, including mental health conditions, are integrated, and identification and treatment of these conditions are conducted at primary health care facilities, with nurses being trained to detect a number of mental illnesses using Primary Care 101 (PC101). PC101 is an integrated set of chronic care guidelines adopted by the national Department of Health that include mental health to assist primary health care staff in the management of multiple and often co-existing chronic diseases. A number of benefits to providing holistic services for integrating mental health care were identified, in addition to a number of challenges.

With respect to the benefits, including mental health as part of the ICDM at primary care level was seen as a way of providing holistic care, increasing access and decreasing stigma. According to a national representative, ICDM would assist in reducing stigma, because mental illness will be seen “as a chronic disease, just like any other disease” (DR2). It was also felt that ICDM would reduce the workload of health professionals. Because the needs of people with any chronic illness are “pretty much the same,…you can use one methodology of long term care across different disease patterns” (NR1). The benefits of the patient-centred care focus of the ICDM model was also emphasised by a district representative: “the client … also giv(ing) their inputs. What are … their needs, what do they want, not what we perceive as what their needs are” (DR4). There was recognition of the benefits of integration in terms of patient outcomes and addressing comorbid conditions. PC101 was viewed as providing the vehicle for integration: “PC101 will really assist us to see that everyone is on board, that they are trained and …well conversant on how to manage a mental health client” (DR4).

With respect to challenges, negative attitudes and lack of experience or training in mental health by primary health care nurses in particular was identified as a major barrier to responsiveness to integrated mental health care, with the integrated model not “be(ing) fully embraced by all health workers” (NR1), and a prevailing belief that psychiatric patients need psychiatric hospitals. Participants identified part of the problem as insufficient involvement of service providers in planning of service provision, leading to lack of buy in: “So I have noticed with some of our clinics that they have actually regressed because it was a talk-down and staff wasn’t really involved in the development of it” (DR5). This may have filtered down from the uncoordinated planning and lack of intersectoral collaboration at national and provincial levels.

Representatives from national, provincial and district levels all suggested that a major challenge relating to integrated chronic disease management was resistance from patients: “There (are) still (mental health) patients who would prefer to be seen separately from other patients because they believe maybe they, or they think they were receiving better quality when they were seen separately” (DR2). “The problem is with the mental health users. They are resistant to integrate at the clinics. They want to have their own rooms…and their only person for consultation” (PR3). “As soon as the ICDM started, patients didn’t want to be integrated with the rest of the facility patients and so they disappeared” (DR3).

A lack of continuity in care also emerged as a challenge. With the integrated model, patients are not guaranteed that they will see the same nurse at each consultation, and some mental health care patients struggle with this. Poor communication between critical departments also emerged as bedevilling continuity of care. An example provided was the poor follow up or tracking of patients for adherence in those admitted to an institution, and then down referred to clinics for follow-up medication, with the result that patients relapse more often. A national representative noted that,

“We are losing many patients or users into the cracks because you find that the user is seen at a facility, maybe admitted. Once the user leaves the facility, we don’t keep track whether the user ultimately follows up, adherence to treatment, all those aspects” (NR3).

Strategies to address these challenges included the need to provide mentoring support and supervision for PC101 in addition to the training in PC101 for primary health care staff. Change management could re-orientate and raise awareness of staff at facilities about the benefits of integration for overall health, while awareness raising in the community could educate service users about the benefits of integration and facilitate buy in.

#### Responsiveness at a community level

With regard to community-based services, the importance of empowering communities to play a greater role in caring for patients at a community level was emphasised. A national representative suggested that, while government could do a lot more, opportunities exist to enlist the support of user organisations to work with families and go “beyond what government can do” (NR2). Communities should be encouraged, he said, to informally address their needs. In addition, community health workers, NGOs and Department of Social Development social workers could also be capacitated to deliver community-based psychosocial services.

With regard to challenges, the move to deinstitutionalisation and introduction of community-based services has been slow, and services are largely still concentrated at institutional level. Because of gaps in the provision of community-based residential care, as well as psychosocial rehabilitation programmes to facilitate recovery and reintegration into the community, many patients relapse and have to be re-admitted to hospital. In other cases, where residential facilities exist, a lack of structured psychosocial programmes at these facilities was identified as a problem. One participant also mentioned possible resistance from families and communities as reflected in the following quotation:

“our approach that we inherited from the previous … apartheid government of institutionalising people with disabilities, has in a way created some dependency and some expectations especially among the family members. And now when you really get and move the services to the community where a large number of people with mental disability can actually then be able to receive the services, we might actually meet with some resistance especially from the community and from families, because communities know that they’ve got this perception and understanding that if you have a mental disability then you should be closed up in an institution” (NR4).

A shortage of human resources is another significant challenge. In the Department of Health (DoH), there is lack of human resources to provide community-based services, so “even if there can be resources in terms of financial resources…there are no warm bodies to actually render service” (NR4). Community health workers are not capacitated to intervene in mental health, and there are no DoH-employed social workers at primary care level. There also seems to be some confusion regarding who is responsible for providing community-based services and a lack of coordination and role clarification between sectors. The result is a gap in services at this level. According to one participant: “they’re concerned about who’s going to run it. Because …they say it’s not a (Department of) Health competency. Because they think Social Development or something should run it” (DR8).

It is thus not surprising that the need to establish collaborative arrangements between the Department of Health, Social Development, Housing and other sectors at national, provincial and district levels, with respect to community-based psychosocial rehabilitation (service provision and funding) was identified as a strategy to address this gap in services by a number of participants, as was the redistribution of resources from tertiary-level institutions to community-based services.

### Effectiveness & Efficiency

With the focus on effective use of resources to meet health needs, the sub themes that were developed in this governance principle included human resources capacity, financing, and infrastructure.

#### Human resources

Capacity barriers focused on the shortage of human resources to implement the mental health policy. The Mental Health Policy Framework and Strategic Plan stipulates that mental health teams need to be established in each district. However, respondents anticipated a number of challenges with establishing these teams. Apart from the existing staff shortages, participants indicated that finding or attracting suitably qualified staff to join the district teams would be difficult, particularly in more remote or rural areas. Some participants were concerned that, even if they could find the specialists to create these teams, the posts had not been created to appoint them within the existing staff structures. Even if the staff structures could be created, participants were concerned that there are no resources to sustain funding these posts, because the money would also have to come from the equitable share budget pool. As suggested by one district level participant:

“If only the staff structure can be addressed whereby these posts are available at sub district level, then it will be easy for sub districts to have appointments. But if it’s not on the staff establishments, then it’s really a challenge to get these people appointed” (DR4).

Suggested strategies to overcome these barriers included diversifying the roles of professionals who are already appointed in the system and using existing resources more effectively. As one national level participant explained,

“some of the provinces are now starting to look at where they can move resources to where they might be more effectively utilised. And, you know, if you start with psychologists, well maybe you can go onto OTs and to social workers and to other resources as well – maybe the nurses” (NR1).

Further, a district level participant explained that, despite expansion of services, additional posts were not being created, which placed greater burden on existing staff: “You continue to expand your services, expand expand expand, with the same staff structure…And that is one of the reasons people are leaving, definitely, burnout and work overload” (DR8). This problem applies to both generalised and specialist personnel, with the few specialists who do work in primary health care, particularly psychologists and psychiatrists, having a very high work load. The need to capacitate general health professionals to identify and treat mental disorders within a task sharing approach was emphasised by a number of participants. As suggested by one participant, the key, is to “capacitate health care providers in terms of identifying mental disorders or mental illnesses, managing them, and also follow up care” (NR3). There was a lot of support among participants for having all primary health professionals trained in PC101. Factors compromising optimal implementation of PC101 included: i) insufficient capacity to provide training and support for PC101, as well as the general lack of clarity regarding responsibility for supervising and monitoring implementation of PC101; and ii) the attitude that mental health was not the responsibility of primary health care providers, coupled with high workloads, leading to poor uptake of PC101. As suggested by a district representative:

“Attitudes are really a difficult thing to change, and you are not always there at the facility. You’ll find when you’re doing monitoring and evaluation, they will manage the client correctly but as soon as you turn your back…but we must also look at the factors why, why are people (not identifying mental disorders), (it is) their attitudes, at times it’s severe workloads, it’s other pressures that we are not considering” (DR4).

Providing staff with training and support was seen as one way to combat the resistance to the integrated care model as well as dealing with high staff turnover. This training needs to be continuous and followed up regularly to ensure that people are following the PC101 guidelines with respect to identifying mental health issues. The potential role of district mental health teams in providing supervision and support was highlighted. There is also a need for greater collaboration with the Department of Education to adapt training towards comprehensive chronic care, and to train more graduates.

The involvement of community health workers and lay counsellors in mental health services was also discussed. In general, participants seemed supportive of the use of community health workers in providing mental health screening and follow up services, and using lay counsellors in the provision of counselling for common mental disorders. However, there were concerns that they do not have adequate training and similar to PC101 do not have continuous support and supervision from specialists. There was also a need for role clarification with respect to the duties and responsibilities of these counsellors.

#### Financing

Some participants believed that funding for mental health is inadequate. This is partly because mental health shares a budget pool (“equitable share”) with other health programmes and “everyone is fighting for the equitable share” (DR5). The existing shortage of resources was identified as being a obstacle for the implementation of the mental health policy. As suggested by a national participant: “A lot of provinces are already overspent. And anything more that you give them (ask them to do) does become a problem…if they (are) starting services from scratch, with the resources that they’ve got, what would they do?” (NR1). There seems to be a disparity between provinces in terms of resource allocation for mental health.

Others highlighted the budgeting process as problematic, with funds being allocated to mental health based on where the funds were allocated the previous year, but with an inflation-related increase. Without tying funds to specific activities, there will be difficulties in operationalising many of the stipulations of the mental health policy. As suggested by a district representative, this historical way that the budget is allocated is

“going to be detrimental to implementing (the policy), it really will. I mean but I think our provincial head office knows that the way they are budgeting doesn’t make sense in today’s world any more, they know they should be getting a health economist in and really do an overhaul but, that hasn’t happened” (DR8).

Strategies to overcome these difficulties included shifting resources from specialized hospitals to community care in the long term, but this was linked to the need for activity-based budgeting. Leveraging resources from other health programmes such as HIV/AIDS, which were better funded, was also suggested as a potential strategy.

#### Infrastructure

Participants spoke about problems with quality and quantity of existing infrastructure to facilitate the shift to community-based care in particular. This was partly attributed to a lack of coordinated planning between sectors: “capital planning was just building hospitals without consulting us” (PR3). The paucity of community or residential centres to facilitate the integration of patients who are discharged from hospitals back into the communities was highlighted. There was also general agreement that there is “a huge challenge in terms of privacy and space” (DR4) for providing counselling services in primary health care clinics. According to one district representative, “they (counsellors) are a bit at the bottom of the rung, so…they don’t really have dedicated space” (DR5) in which to see patients. Possible ways of addressing this problem identified included looking creatively at how to make more counselling space available. In particular, participants spoke about making use of park homes to add space to primary health care facilities and provide dedicated space for counselling services. This was also felt to be a less expensive option than building new clinics or adding onto existing infrastructure.

#### Medicines & technologies

Problems with the consistent supply of psychotropic medication at district level also emerged as a major challenge. As indicated by a participant at district level:

“There is a tender process. There is a supplier that is contracted in and there are sometimes challenges that we will experience with suppliers not being able to supply on time or the adequate amounts and it impacts directly (at the) operational level. Clients will come to the facility and supplies wouldn’t be there, which again contributes to them defaulting” (DR1).

Another challenge concerned communication problems between different health care providers, particularly between hospitals, clinics and pharmacies. In some instances, the correct medication was not provided because of human error. In other instances, there was a breakdown in communication between the facility and the hospitals which were down-referring patients, as well as the facility and the pharmacy, where (in the latter case) the “script isn’t written or sent on time, the medication isn’t packed on time to be available for the patient, and so the patient defaults” (DR4). A breakdown in communication was also identified as a problem that needed to be rectified with respect to drug prescriptions and delivery systems. In addition, difficulties in ensuring adequate availability of the PC101 manuals in primary health care facilities was identified as a hindrance to the implementation of the PC101 guidelines. Having a master file in each facility and then seeing what could be made available in each consulting room was identified as a potential strategy to address this problem.

### Participation & Collaboration

Originally participation and consensus orientation, this principle focused on the participation of stakeholders in decision making in health. We expanded this definition to include the collaborative planning and decision making that underscores effective service provision.

Participants were asked about whether the Department of Health (DoH) engages in consultation with other sectors, particularly the Department of Social Development (DSD), regarding the provision of psychosocial rehabilitation and community-based services. The majority of responses concerned the lack of collaboration and delineation of roles between the two departments, with one provincial participant describing the contact between the two departments as “erratic or not really organised” (PR4). From a national perspective, variation in the degree of collaboration between provinces and districts was highlighted as indicated by a national participant:

“There are areas that are already involved with Social Development in terms of providing community-based residential and day care services, including services for children with intellectual disabilities…but in other provinces and districts, Social Development doesn’t really play the role that is prescribed in that plan” (NR3).

A general problem regarding collaboration across all sectors was the lack of coordination due to different roles and mandates, such that collaboration does not filter down from planning to implementation level: “Provincial health has got to get cooperation from other departments…so it becomes much more complex because of the different mandates that the different departments have” (NR2). This seems to be compounded by the reluctance of some departments to get involved in the implementation of mental health policies and legislation. In relation to strategies to address the problem of collaboration, the need for a memorandum of understanding between departments, particularly the Department of Health and Department of Social Development, was suggested to assist with formalising collaboration and clarifying roles and responsibilities with respect to mental health. A national representative spoke about the establishment of the National Health Council, which would formalise collaboration between sectors. However, one participant felt that discussions around collaboration actually usually take place at operational level, but what was needed was

“this kind of collaboration to take place at all levels, whereby even the executive managers, like heads of department, are to meet and talk about particular issues, they need to do that so that they can agree to say, for our Department this will be the way forward. So that when the implementers come in, it’s not about them having to pave the way forward for how they are going to work, it should have been cleared at a high level” (PR4).

This would require capacity building and commitment at leadership level to build stronger partnerships, as well as building capacity among health professionals and managers to advocate for mental health within their programmes and departments.

There was also acknowledgement that consultation with service providers and service users was not adequate. “Even the involvement of service, you know, other service providers, the private sector, users themselves, families themselves, it is very poor” (NR2). As a result of this lack of involvement, there is some resistance to policy directives among service providers. There was no question among participants about the need for greater involvement of families and service users both in policy development and service planning, and in treatment decisions. However, there was uncertainty regarding how to increase involvement: “we regard that as an important element, but it has not as yet happened. And we are not so sure even in terms of the modus operandi, how to achieve that” (PR4). Capacity building in stakeholder engagement was also suggested as a strategy to address this problem as indicated in the following quotation:

“I was talking to a Prof from the University…and he said, but yeah but first of all you have to train people how to do local engagement! I said why? He said no, people don’t automatically know how to do this, you know, we assume, it’s just talking but it’s not just talking” (DR8).

Service users could also be consulted through clinic committees and advocacy groups, as well as through holding *imbizos* (gatherings) to get community input. This would require building capacity of service user groups and allowing for formal inclusion in collaborative structures.

### Equity & Inclusiveness

Originally equity, we expanded this principle to emphasise inclusiveness, as we considered this critical to ensuring equal opportunities to improve or maintain health. In South Africa, the notion that all men and women should have equal opportunities to improve or maintain their mental health and well-being centred on two main issues: access to services and stigma.

Responses about access to services were a mixture of positive and negative perceptions. On the one hand, participants spoke about integrated care as a mechanism for increasing access to services for mental health care users. There was emphasis, from participants at national level, on the plans for increasing access to services, such as public education programmes, help lines and allocating funds to improve access for families and communities and combat the lack of awareness among service users regarding how and where to access mental health services. Responses from provincial and district level participants, however, focused more on the challenges associated with service provision, which limit accessibility. One issue is the geography of the North West Province, which is a “vast and rural province. So we’re still not at a point where we can say, look, services are, you know, readily accessible” (PR2), with mental health services concentrated in the urban areas. There is also disparity between districts in terms of the number of facilities and community centres, and a lack of qualified staff to provide mental health services, both of which were identified as barriers to access.

With regard to stigma, respondents suggested that the mental health policy framework does not provide sufficient guidance on how stigma should be addressed, with provincial and district level respondents, not being aware of any specific anti-stigma programmes for mental health and variations between provinces in terms of prioritising addressing stigma. Furthermore, there is a shortage of staff to drive these programmes. However, integrating mental health into primary health care services, and particularly chronic care, was seen as having the potential to reduce stigma. Support from provincial and district managers was seen as a facilitative factor in the implementation of anti-stigma programmes. Suggestions for strategies to reduce stigma in communities included campaigns, mass media and the use of testimonials of role models to create awareness and improve mental health literacy. It was suggested by one participant that peer educators or people of the same age and gender would be best suited to convey anti-stigma programmes because “for me, most children or adults better listen to people around that they know” (DR3). The importance of targeting all levels, including the community, employers, service providers and the patients themselves, who have internalised or “self imposed stigma” (DR2) was mentioned by a number of participants. Support groups were seen as important forums for empowering service users.

### Ethics & Oversight

We redefined this principle as ethics and oversight (originally ethics) to operationalise ethical principles across treatment and research. We considered that the ethical treatment of mental health care users in the provision of services was contingent on ensuring that the quality of services was closely monitored, and that safeguards were in place to protect against unethical treatment and redress grievances. Conducting ethical research with mental health care service users also necessitated that there be safeguards against unethical research. Thus, quality assurance, addressing grievances, monitoring services and safeguarding the ethical conduct of research were major issues considered under this principle.

The Office of Health Standards Compliance is an independent body which is responsible for ensuring quality in all health services, including mental health. These core standards “prescribe that each health establishment or health facility must display the patients’ rights charter and also what type of mechanisms should be followed in terms of lodging a grievance or complaint” (NR3). According to a district representative, “there is a compliments and complaints mechanism in the Department of Health” (DR1) which seems to be replicated at each level, from national down to facility level. However, some felt that more could be done, such as setting up hotlines as part of the Office of Health Standards Compliance, and introducing QualityRights – a WHO programme for inpatient and outpatient psychiatric and care facilities – to all facilities. A participant at district level acknowledged that, while there are sometimes quality improvement projects and while NGOs occasionally help with quality assurance, “it’s not happening on a regular basis” (DR6). Part of the problem was seen to be a lack of indicators against which to evaluate performance and quality.

The Mental Health Review Boards also have a role to play in ensuring ethical treatment and service user satisfaction. However, there is variation between provinces in terms of the effectiveness of the Mental Health Review Boards. As suggested by a national representative, some provinces “are really not doing so well in terms of the establishment of the Mental Health Review Boards. Some boards are not even complete” (NR3), while some Mental Health Review Boards are not functioning “as well as they should” (NR1), because of lack of budget and staff to carry out inspections and follow up on grievances. The disparity between provinces in terms of the functionality and effectiveness of Mental Health Review Boards needs to be addressed.

There were very few responses to questions about what safeguards against unethical research were in place. This seems to be because the process for reviewing and approving research with mental health care users is seen to be the mandate of the research units at national and provincial levels. As one provincial representative said, “that comes down to the research approval process and the process in various ethics committees” (PR1), while another noted that “we’ve got a unit in the provincial office. You know, every sort of research you want to do, it must go via the committee for permission. So nobody can just walk in and just start doing research” (PR2). The National Health Act was also highlighted for the guidance it provides on procedures for conducting research with health care users.

### Intelligence & Information

With its emphasis on information, a major focus of this principle was on monitoring and evaluation, and the associated barriers and facilitative factors. The adequacy of mental health indicators and the mental health information system were also explored.

The necessary structures for monitoring and evaluation seem to be in place, although many felt that the indicators for mental health were not sufficient. As indicated by a national representative,

“we would request more indicators and data to be added. But we are competing with other programmes. And the health information system unit indicated to us that they are planning at reducing the number of indicators for all the programmes….it’s not convincing for them that we do use the indicators at all the levels” (NR3).

According to one participant, one reason for this could be that the district health information system is “under the custody of a different unit” (PR4) and so, to “avoid inundating it with information” (PR4), mental health programme managers need to make a strong case for why a particular indicator should be included on the system.

A number of challenges associated with monitoring and evaluating (M&E) mental health were raised by participants. In addition to the poor quality and quantity of mental health indicators included on health information systems, inadequate human resources to carry out monitoring and evaluation also emerged as an issue. It was suggested that provincial and district officials need to play a greater role in monitoring mental health services. Building M&E capacity at all levels was necessary to improve the use of indicators to inform policy and service planning.

Some participants expressed concern about the amount of information that is collected that sits in the system and is not pulled together to “get a picture of what is happening in a district or province” (NR2). The quality of indicators captured, and their ability to accurately reflect quality of services, was also a concern: The DoH has so many indicators that are “absolutely meaningless”….There are cases where “quality is useless but indicators are just put there to make you feel better” (PR1). Some spoke about the difficulty of establishing indicators for mental health because there are not easily measurable outcomes on which to base them: “If you can think of an indicator that will definitely will be a barometer of how care is happening, that would be fine. But that will take a different thought process to do that. I can’t suddenly come up with one” (PR1). It is thus critical to include indicators for mental health in the health information system that provide sufficient information to inform intervention decisions and assess quality improvements.

### Accountability & Transparency

These two principles were relatively under-developed and did not get a lot of responses from all participants at national, provincial and district level. Rather than this being an indication of poor accountability and transparency processes or mechanisms, it is more likely that the questions around accountability and transparency were frequently not asked. Some of the issues could be argued to have been covered in other sections, particularly around monitoring and evaluation.

A comprehensive overview of the challenges, facilitators and recommendations pertaining to each of the governance principles is provided in Table 2 below.

**Table 2:** **Mental health system governance challenges, facilitators & recommendations: overview**

**Principle**	**System level**	**Sub-theme**	**Challenges**	**Enabling factors**	**Recommendations**
**Rule of Law**	Governance	Legislation	Mental health care act lacks guidance on people with disabilities	Synergy between mental health care act & mental health policy	Clarify roles & responsibilities with respect to the act, particularly across sectors
		Enforcement	Insufficient training affects compliance & implementation		Provide sufficient training on mental health legislation & policies
**Strategic Direction**	Governance	Development of policies & plans	Lack of communication about the policy at district level	Including strategic planners in development of plans	Build capacity to translate policies into plans at provincial and district levels
			Insufficient capacity to translate policies into plans due to shortage of staff and skills	Champions who can advocate for mental health Support from national office	Include strategic planners in development of mental health plans
				District mental health teams used as a unit for planning	Use district mental health teams as a unit for planning at local level, provided they are sufficiently capacitated and supported
		Implementation of policies & plans	Poor coordination in terms of planning & service provision between national, provincial & district levels	Clear understanding of roles & responsibilities with respect to implementation	Capacity building of managers in change management to facilitate the implementation of integrated collaborative chronic care, including mental health
			Disparity between provincial mental health units in terms of capacity	Coordination between different stakeholders	Clarify roles & responsibilities of different stakeholders & improve coordination
			Lack of qualified managerial staff to push implementation at ground level		Address resource and capacity disparities between provinces
			Insufficient budget & inadequate infrastructure		
**Responsiveness & Integration**	Governance	Prioritisation of mental health	Mental health still not a priority in the face of many other health needs	Drive by national to develop policy seen as a step towards prioritisation of mental health by national government	Providing training and support in PC101 can facilitate integration of mental health into primary health care
			Mental health seen as separate from other health needs		Education & awareness raising about the benefits of integration among service providers & service users could facilitate buy-in
	Service Delivery	Integration at facility level	Uncoordinated planning & lack of intersectoral collaboration hinders integration Negative or misinformed perceptions about mental health and integration Insufficient involvement of service providers in planning, leading to lack of buy in Lack of training on mental health among health professionals & lack of patient-centred orientation Inadequate follow-up between primary care facilities and tertiary institutions Resistance from mental health care users	PC101 can facilitate integration Recognition of benefits of integration in terms of patient outcomes & addressing comorbid conditions	Establish collaborative arrangements between the Department of Health, Social Development, Housing and other sectors at national, provincial and district levels, that establish clear roles and responsibilities with respect to community-based psychosocial rehabilitation (service provision & funding) Redistribute resources from tertiary-level institutions to community-based services
		Integration at community level	Services still concentrated at institutional level Lack of coordination and role clarification between sectors Shortage of community-based centres & poor accessibility Shortage of human resources to deliver community-based services Resistance from families & communities	Redistributing resources from hospitals to communities Utilising DSD social workers, community health workers and NGOs in delivery of services, but need to be sufficiently capacitated Committed leadership driving this	
**Effectiveness & Efficiency**	Human resources	Human resources capacity	Shortage of health professionals & specialists to implement policy High workload and high staff turnover Inflexibility of existing staff structures to accommodate creation of new posts for district mental health teams Budget not sufficient to appoint more staff Negative attitudes and resistance among staff to treating mental health	Building staff confidence & competence to treat mental health Creation of district mental health teams facilitated by using existing systems Flexibility & using existing resources more efficiently could facilitate establishment of district teams Adapting training to be more primary health care focused Entering into agreements with local universities to train graduates	Given shortage of mental health specialists, particularly in rural areas, need flexibility in creation of district mental health teams (e.g. pooling resources across districts) Collaborate with Department of Education to adapt training and train more graduates An orientation to comprehensive care and change management is needed
		Task sharing	Insufficient specialist capacity to provide training and support in PC101	In-service, on-site & continuous training for health professionals	Task sharing can relieve pressure on health professionals
			High workloads mean poor uptake of PC101 Lack of clarity regarding responsibility for supervising & monitoring implementation of PC101	District mental health teams could provide supervision & support Community health workers, home-based care workers and ward-based outreach teams to provide screening & follow up Role clarification for counsellors to include mental health	PHC personnel trained in PC101 need mentoring and support in implementation of mental health aspects Use lay counsellors as they will relieve pressure on health care professionals, but provide adequate role clarification, training and supervision Use community health workers, home-based care workers and ward-based outreach teams for screening, referral and follow up
	Financing	Financing	Funding for mental health is inadequate Disparity between provinces in terms of resource allocation for mental health Historical budget allocation is problematic	Using existing resources more efficiently – phased approach and piggy backing onto other programmes	Use existing resources more efficiently through, for e.g. a phased approach and piggy-backing onto other programmes Revise way of budgeting from historical to activity-related allocation of funds
	Infrastructure	Infrastructure	Quantity and quality of existing infrastructure not sufficient Lack of coordinated planning between relevant sectors Lack of adequate counselling space in primary care facilities Breakdown in communication between hospitals, clinics and pharmacies results in inconsistent provision of medication Inadequate availability of PC101 guidelines	Creative ways of making more counselling space available – e.g. gazebos and park homes Extra steps taken to ensure patients get medication (e.g. delivering to patients homes) Master file of guidelines available at facilities	Include planning for counselling space within PHC facilities Improve communication between clinics, hospitals and pharmacies with respect to drug prescriptions and delivery systems Ensure availability of master file of protocols and guidelines in each facility
**Participation & Collaboration**	Governance	Inter-sectoral	Lack of coordination & collaboration between sectors due to different roles and mandates Coordination does not filter down from planning to implementation level Reluctance of some departments to get involved in implementation of mental health policies & legislation		Clarify roles & responsibilities of different departments with respect to mental health Build capacity & commitment at leadership level to create stronger partnership between DSD & DoH; formalise structures to improve collaboration Train managers in stakeholder engagement
		DoH – DSD	Lack of coordination in terms of planning & provision of psychosocial rehabilitation services Lack of clarity of roles and mandates	Capacity building & commitment at leadership level could help to build stronger partnership NGOs are the implementing arm of DSD – DoH could work through them	Build capacity among health professionals and managers to advocate for mental health
	Governance	With service users & service providers	Inadequate consultation with service providers Some resistance to policy directives among service providers	Service users consulted through clinic committees and advocacy groups and through holding *imbizos* to get community input	Improve consultation with service users through service user groups and communication with caregivers
			Uncertainty about how to best consult with service users Need for greater involvement of families & service users in treatment decisions		Build capacity of service user groups to engage in advocacy, and allow for formal inclusion in collaborative structures
**Equity & Inclusiveness**	Governance	Access	Size & remoteness of some provinces & districts make access to services difficult Disparity between districts in terms of number of facilities and community centres Lack of qualified staff to provide mental health services a barrier to access	Integrated care increases access Public education programmes a means to increase awareness; helplines a means to increase access	Integrating mental health into primary health care could increase access Raise awareness among service users regarding how and where to access services
		Stigma	Policy framework is not clear on how to address stigma Disparity between provinces in terms of how stigma is addressed Shortage of staff to drive these programmes Negative perceptions, driven by ignorance, lack of awareness and fear, a barrier to reducing stigma	Integrated care could reduce stigma Support from provincial and district managers could facilitate implementation of stigma programmes Using different forms of media to reach communities Support groups can empower users	Integrating mental health into primary health care could help to reduce stigma Implement anti-stigma campaigns in the community, with support from district and provincial managers Mass awareness campaigns using different forms of media, role models and support groups to reach and empower service users; clarify whose responsibility it is to do this
**Ethics & Oversight**	Governance	Ethical treatment	Disparity between provinces in terms of functionality & effectiveness of Mental Health Review Boards Staff shortages a hindrance to carrying out inspections & following up grievances Lack of indicators against which to evaluate performance	There are a number of mechanisms for ensuring quality/standards in health services in general, applied to mental health	Address disparity between provinces in terms of functionality and effectiveness of Mental Health Review Boards Introduce the WHO Quality Rights project and capacitate Mental Health Review Boards to use the toolkit to ensure that standards are being met
		Ethical research		Research units and ethics committees at provincial and national levels oversee health research National Health Act provides guidance on procedures for conducting research with health care users	
**Intelligence & Information**	Information		Lack of monitoring mechanisms/systems at all levels Indicators for mental health in the health information system are not sufficient in terms of quantity or quality Inadequate human resources to carry out M&E		Provincial and district officials need to play a role in monitoring quality of mental health services Build M&E capacity at all levels and improve the use of indicators to inform policy and service planning
					Include indicators for mental health in the health in the health information system that provide sufficient information to inform intervention decisions and assess quality improvements

### Discussion & conclusions

The application of the analytical framework to assess mental health system governance at the national, provincial and district levels in South Africa has revealed some positive elements, and a number of areas requiring intervention. The challenges identified at governance level are reflective of the challenges encountered at implementation level, suggesting that strengthening aspects of the health system – such as human resources and infrastructure – could enhance governance. The weaknesses were particularly in relation to the governance principles of strategic direction, responsiveness & integration, effectiveness & efficiency, and participation and collaboration. In particular, the findings suggested a strong need for: i) capacity building at all levels of the health system, ii) greater coordination and collaboration in planning and service provision, iii) consultation with stakeholders, iv) training and supervision in PC101, v) infrastructural improvements, vi) streamlined delivery systems of drugs and treatment protocols, vii) implementation of quality improvement programmes, viii) better indicators for mental health in the health information system and ix) advocacy and awareness raising campaigns. Despite having a new mental health policy that supports integration of mental health into primary health care, in South Africa, as elsewhere, a weak health system and health system governance make it difficult to implement this policy and deliver cost-effective interventions for scaled up mental health care [37,38]. Based on the findings of the current study, recommendations are provided for improving health system governance to support integrated mental health in South Africa.

Mental health legislation, policies and plans are needed to integrate mental health into primary health care [10]. South Africa has a Mental Health Care Act which has been in place since 2002. The findings of this study suggest that, although this Act has a strong human rights focus and is generally viewed positively, insufficient training has affected compliance and implementation of the Act, as has poor coordination between sectors. The Rule of Law principle requires that legal frameworks pertaining to mental health should be fair and impartially enforced. In order to improve governance of this aspect, it is recommended that further training be provided on the enforcement of the Mental Health Care Act and that all departments responsible for enforcing the Act be included in this training. Similarly, there seems to be a lack of capacity to push implementation of the new mental health policy framework at ground level. The Strategic Direction principle is based on the notion that leaders should have a “broad and long-term perspective on (mental) health and human development, along with a sense of strategic direction for such development” [30]. Although the South African mental health policy framework seems to capture this vision and provide such strategic direction, findings here suggest that there is a need for capacity building to translate plans at provincial and district levels, consistent with studies suggesting that sound mental health policies or legislation do not necessarily translate into improvements in services at local levels [39]. Building capacity to implement policies and plans would include using the new district mental health teams as a unit for planning at local level, as well as training managers in change management for the implementation of integrated chronic care, including mental health. The capacity and resource disparities between provinces also need to be addressed in order for the policies and plans to be effectively operationalised. We would add to these principles the WHO [10] guideline that integration is a process, not a once-off event. Thus, implementation of the policy towards integrated mental health care will take time and a concerted effort to secure buy in through involving all stakeholders in the process.

The principle of Responsiveness captures the idea that institutions and processes in the health system should serve all stakeholders and ensure that the policies and programmes that are put in place are responsive to the needs of all health care users. Because integration is fundamental to adapting the health system to respond to the needs of mental health care users, we amended this principle to Responsiveness and Integration. We found that mental health is still not prioritised across various levels of the health system, and is still seen as separate from other health needs. There is resistance on the part of primary health care providers and users and their families to integrated care at both facility and community levels. As in other areas, there is also uncoordinated planning and poor intersectoral collaboration and role clarification. Services are still concentrated at institutional level, and there is a shortage of community-based services. Service providers are also not sufficiently trained in dealing with mental health issues and continue to be trained in a biomedical orientation towards acute care, rather than holistic, patient-centred care. Thus, although South Africa is committed to moving towards integrated mental health care, there are a number of systemic factors that hinder the realisation of the principle of Responsiveness and Integration. This can be improved by establishing collaborative arrangements between the Departments of Health, Social Development and other sectors that outline clear roles and responsibilities, particularly with respect to community-based psychosocial rehabilitation. There is a need to redistribute resources from tertiary level institutions to community-based services, which would require the provision of training and support in PC101 to facilitate integrated care. This is echoed by Petersen et al. [40], who suggest that the injection of mental health resources into primary health care and more efficient use of existing resources is needed to achieve deinstitutionalisation and comprehensive integrated primary health care in South Africa. In addition, education and awareness raising about the benefits of integration among service providers is likely to facilitate buy-in to integrated chronic care for mental health.

Making the best use of resources while ensuring that processes and institutions in the health system meet population health needs underscores the principle of Effectiveness & Efficiency. Here, health system building blocks of human resources, financing and infrastructure come into play, as well as the provision of essential drugs and treatment protocols. A number of challenges relating to human resources for integrated care were identified in this study. These included a shortage of health professionals and specialists to implement the policy and insufficient budget to appoint additional staff; inflexible staff structures limiting the creating of new posts for district mental health teams; and poor uptake of PC101 and limited capacity for supervision and monitoring of PC101 implementation. There is a need for flexibility in the creation of the district mental health teams, including adapting existing staff structures to create the relevant posts. Pre-service training of PHC nurses should include PC101, while existing staff need training and mentoring in PC101 to support the implementation of mental health aspects. The importance of training primary health care workers in the recognition and treatment of mental disorders and ensuring they have adequate supervision and support has been recognised elsewhere [4,13]. Furthermore, task sharing should be promoted to extend mental health services at facilities by using lay counsellors and in communities by using community health workers. This builds on previous research that task sharing can be effectively extended to lay health workers, particularly when interventions are simplified and proper supervision is provided [3,41,42]. It is thus critical that these non-professionals receive adequate training, role clarification and supervision in order to effectively carry out their duties.

There was widespread recognition in this study that funding for mental health is inadequate, which echoes research showing that there is a gap between the burden of mental illness and the budget allocated to it [43]. This is exacerbated by disparities between provinces in terms of resource allocation. In order to improve governance through Effectiveness & Efficiency, therefore, it is recommended that existing resources be used more efficiently – for example, through a phased approach to policy implementation, or by piggy backing onto other programmes. Maximising the effectiveness of limited resources by reducing redundancies as well as making use of other programmes has been suggested elsewhere [4]. Participants in this study also believed that revising the way that budgeting is done in the Department of Health from an historical to an activity-based allocation, would help to direct more resources towards mental health. Governing in an effective and efficient manner to integrate mental health into primary health care is further constrained by the inadequate quantity and quality of existing infrastructure for mental health service provision. This continues to be identified as a weakness in the response of the health system to the provision of mental health care [44]. It is critical for planning to include counselling space within primary health care facilities in order to shift from a biomedical to a psychosocial model of care. Evidence based psychosocial interventions to treat, promote health and prevent illness need to be taken seriously and included in infrastructure plans. This is important for recovery, as opposed to just providing symptom management which is currently the dominate practice with regards to chronic illness and mental illness [45]. In terms of consistent provision of medication, there is a need to improve communication between clinics, hospitals and pharmacies with respect to drug prescriptions and delivery systems. Previous research has shown that irregular supply of medication remains a challenge in many low- and middle-income countries [39], and requires strengthening of supply chain management systems and training of health care staff [6]. If patients do not receive their medication timeously, they are at risk of relapsing, which has a knock-on effect for the effectiveness of the service. Similarly, making a master file of treatment protocols and guidelines available in each facility could contribute to adherence to PC101 and the holistic treatment of patients for all their health needs. Integrating psychosocial assessments and interventions into management protocols can further assist with promoting a holistic model of care [4].

Due in part to the varied nature of the social determinants of mental health, mental health is essentially an intersectoral issue [6]. Moreover, “all men and women should have a voice in decision making for health…(while) good governance of the health system mediates differing interests to reach a broad consensus on what is in the best interests of the group” [30]. The principle of Participation and Collaboration highlights the need to involve all stakeholders in planning and provision of services, consistent with recommendations that effective integration requires collaboration with other government non-health sectors, nongovernmental organisations, community health workers, service users and communities [10,46]. Findings from this study indicate that this is an area of weakness in the health system in South Africa. There is a lack of coordination and collaboration between sectors due to different roles and mandates, which filters down from planning to implementation level. There is thus a need to clarify roles and responsibilities of different departments with respect to mental health. It is also recommended that capacity and commitment be built at leadership level to create stronger partnerships with other sectors and to formalise structures to improve collaboration. Part of this may entail training managers in stakeholder engagement, as well as building capacity among health professionals and managers to advocate for mental health and overcome the resistance of some departments to get involved in the implementation of mental health legislation and policies. It was also evident in this study that there is inadequate consultation with service providers regarding the development and implementation of policies and plans, resulting in resistance to policy directives. To overcome this, mechanisms for improving participation of service providers in planning need to be improved. In this study, as elsewhere, mental health care users and their families are seldom involved in planning and decision making about mental health service provision, from a macro or policy level [39,47] to a micro or treatment plan level [48]. There is a need to improve consultation with mental health service users through user groups and communication with caregivers, and allowing for formal inclusion in collaborative structures. Service users’ capacity to engage in advocacy should also be strengthened.

The Equity and Inclusiveness governance principle is based on the notion that “all men and women should have equal opportunities to improve and maintain their health and well-being” [30]. In South Africa, this is operationalised as improving access to mental health services, and reducing individual and institutional stigma around mental health issues. Findings showed that the size and remoteness of some areas can make it difficult for users to access services. In addition, the shortage of facilities and community services, as well as the number of staff to provide these services, was considered a barrier to access. It is believed that integrating mental health into primary health care – the crux of South Africa’s mental health policy – could increase access, as well as serve to decrease stigma. Raising awareness among service users regarding how and where to access services is also recommended. However, it has been suggested that the reluctance of service users to access mental health services is partly affected by stigma and discrimination [43]. Because the mental health policy is not clear on how to address stigma, there is a need to implement anti-stigma campaigns in the community, with support from district and provincial managers. It is recommended that mass awareness campaigns use different forms of media, role models and support groups to reach and empower service users. Advocacy has also been recognised as critical to the success of integrated mental health care [10].

Safeguarding the interests and rights of mental health care users and applying the ethical principles of autonomy, beneficence and justice are fundamental to the principle of Ethics and Oversight for good governance. In this study, it was found that there is disparity between provinces in terms of the functionality and effectiveness of Mental Health Review boards, which needs to be addressed. There is also a lack of staff to carry out quality checks and follow up on grievances, as well as a lack of indicators against which to evaluate performance. It is recommended that the WHO Quality Rights project be introduced and Mental Health Review Boards be capacitated to use the toolkit to ensure that standards are being met. The WHO QualityRights Project has objectives of improving the quality of care and human rights conditions in mental health and social care facilities; changing attitudes and building capacity in service users, families and health workers to understand and promote human rights and recovery; promoting the involvement of people with mental disabilities in advocacy work; and reforming national policies and legislation in alignment with best practice and international human rights standards [49]. The QualityRights Project has a toolkit to assist countries to assess and implement strategies to meet key standards in inpatient and outpatient mental health and social care facilities, which are in alignment with the International Convention on the Rights of Persons with Disabilities. Implementing this toolkit is likely to enhance governance through the principle of Ethics and Oversight, as well as improve Participation and Collaboration efforts through empowering service users to advocate on their own behalf.

Finally, “intelligence and information are essential for a good understanding of the health system, without which it is not possible to provide evidence for informed decisions” [30]. Being able to systematically gather and assess information about mental health issues is fundamental to the principle of Intelligence and Information. However, results of this study suggested that there is a lack of monitoring and evaluation mechanisms at all levels of the health system, as well as inadequate human resources to carry out monitoring and evaluation. Indicators for mental health in the health information system are also not sufficient in terms of both quantity and quality. This is consistent with other findings that mental disorders or health are not adequately captured in routine health information management systems in most low- and middle-income countries [6] as well as in South Africa [44]. It is thus recommended that monitoring and evaluation capacity be built at all levels, to improve the use of indicators to inform policy and service planning. In addition, provincial and district officials need to play a role in the monitoring of the quality of mental health services through the systematic use of information. Further, it is critical to include indicators for mental health in the health in the health information system that provide sufficient information to inform intervention decisions and assess quality improvements.

## Abbreviations

DOH: Department of Health
DSD: Department of Social Development
ICDM: Integrated Chronic Disease Management
LAMICs: Low- and middle-income countries
MHPF: Mental Health Policy Framework
NCDs: Non-communicable diseases
PC101: Primary Care 101
